# Association between anthropometric criteria and body composition among children aged 6–59 months with severe acute malnutrition: a cross-sectional assessment from India

**DOI:** 10.1186/s40795-022-00551-6

**Published:** 2022-06-23

**Authors:** Rajesh Kumar Sinha, Praveen Kumar, Abner Daniel, Hemang Shah, Raja Sriswan, Arun Kokane, Aditya Mohapatra, Vivek Kashyap, Anil Kumar Goel, Virendra Kumar, Asha Kiran, N. Arlappa, Ankur Joshi, Rashmi Ranjan Nayak, Shikha Sayal, Arjan de Wagt

**Affiliations:** 1 National Centre of Excellence for Management of Children with Severe Acute Malnutrition (NCoE-SAM), Kalawati Saran Children’s Hospital, C-604 Connaught Circus, DIZ Area, Connaught Place, New Delhi, 110001 India; 2 Lady Hardinge Medical College and associated Kalawati Saran Children’s Hospital, C-604 Connaught Circus, DIZ Area, Connaught Place, New Delhi, 110001 India; 3 UNICEF India Country Office, 73, Lodi Estate, New Delhi, 110003 India; 4 Children’s Investment Fund Foundation, The Crescent, Level 3, Lado Sarai, New Delhi, 110030 India; 5 ICMR-National Institute of Nutrition, Beside Tarnaka Metro Station, Osmania University, Hyderabad, PO Telangana-500007 India; 6 All India Institute of Medical Sciences, Bhopal Saket Nagar, AIIMS Campus, Saket Nagar, BaghSwaniya, Bhopal, Madhya Pradesh-462020 India; 7 Addl Director, Department of Health and Family Welfare, Government of Odisha Annex Building, SIHFW, Bira Maharana Ln, Nilakantha Nagar, Nayapalli, Bhubaneswar, Odisha-751012 India; 8 Rajendra Institute of Medical Sciences, Ranchi, Jharkhand-834001 India; 9 All India Institute of Medical Sciences, Raipur Gate No, 1, Great Eastern Rd, opposite Gurudwara, AIIMS Campus, Tatibandh, Raipur, Chhattisgarh-492099 India; 10 Joint Secretary, Department of Women and Child Development and Mission Shakti, Government of Odisha, Mission Shakti Bhawan, At-Gandamunda, PO-Baramunda, Bhubaneswar, Odisha Pin-751030 India

**Keywords:** Severe acute malnutrition (SAM), Weight-for-height z-score (WHZ), Body composition, Fat mass, Fat free mass

## Abstract

A multicentric study is being conducted in which children with severe acute malnutrition (SAM) aged 6–59 months are identified with only weight-for-height z-score (WHZ) < − 3 criteria. The present study aimed to assess associations of anthropometric parameters and body composition parameters, to improve treatment of SAM. We conducted a cross-section assessment using the enrolment data of children who participated in a multi-centric longitudinal controlled study from five Indian states. Fat-free mass (FFM) and fat mass (FM) were determined by bio-electrical impedance analysis (BIA). Six hundred fifty-nine children were enrolled in the study using WHZ < -3 criteria. Available data shows that WHZ, WAZ and BMIZ were significantly associated with FFMI while MUACZ was significantly associated with both FMI and FFMI. Children with both severe wasting and severe stunting had significantly lower FFMI compared to those who were only severely wasted. All forms of anthropometric deficits appear to adversely impact FFM and FM.

**Trial registration**

The study is registered with Clinical Trial Registration of India (Registration No.: CTRI/2020/09/028013 dated 24/09/2020).

## Background

Different countries are making substantial progress in prevention and treatment of child undernutrition, which remains a major global public health challenge and still contributes to around half of all deaths in children under 5 years of age [1]. Greater understanding on association between undernutrition using anthropometric parameters and body composition parameters such as fat mass (FM) and fat free mass (FFM) might enable the development of more effective clinical and public health interventions towards better survival and quality of life. Generally, undernutrition among children is usually assessed using anthropometric measurements (height and weight) [2] and categorising undernutrition among children as either ‘stunted’ (low height for age z-score, HAZ), or ‘wasted’ (low weight for height z-score, WHZ) or ‘underweight’ (low weight for age z-score, WAZ) [3].

The physical consequences of undernutrition can be further understood through body composition analysis which divides body mass into FM and FFM [4]. The assessment of nutritional status using anthropometry is a proxy indicator for changes in body composition, which are more complex to measure but they are better indicator of energy reserves [5]. The amount and distribution of FM and FFM, are associated with functional organs and tissues among children and also important for immune function [5]. When a child recovers from malnutrition, it is important to re-establish both FM and FFM. However, we still don’t know to what extent each is required to re-establish. Generally, we take weight gain as a sign of nutrition recovery. However, weight gain does not show to what extent FFM is increased, as weight gain following severe illness has been seen to be gains in FM rather than FFM [5]. It is thus important to assess change in body composition parameters alongside anthropometric parameters such as weight gain.

Each of these components is of interest but potential effects of undernutrition on FFM have received little attention. When a child has insufficient dietary protein, muscle mass may provide critical proteins for immune function. Depleted muscle mass is a risk factor of mortality [4, 5]. Similarly, FM provides energy for immune function and secretes leptin [6, 7]. Studies have shown that low level of leptin causes child mortality [8, 9]. In the recent times, measuring body composition of malnourished children to understand any long-term consequences of treatment provided to them has become an important research topic [10–12]. Concerns have also been raised that malnourished children treated with energy dense diet have risk of a disproportionately greater deposit of fat which poses the long-term risk of obesity and chronic metabolic disease in their later stage of their life [10].

Different studies have found varying associations between anthropometric and body composition parameters. Studies in India and Senegal have found direct associations of stunting with FM [13, 14], while no associations were found among children in Jamaica, Peru or South Africa [15–18]. A study with stunted adolescents aged 11–15 years in Brazil have found that compared to girls, boys gained more FM [19]. The present study is a baseline analysis of an ongoing multicentric longitudinal, controlled study, assessing the effect of government implemented community-based management of children with uncomplicated SAM providing locally prepared nutrient dense food in five Indian states [20]. The specific objectives of the present study are:

- To determine associations between anthropometric and body composition indices among studied children at baseline.
- To determine whether anthropometric and body composition indices are different between different groups such as age, sex, caste or household wealth index.

The findings can aid in better understanding of associations between body composition indices and other socio-demographic factors alongwith nutritional parameters defined using anthropometric indices. Understanding the relationship between anthropometric and body composition indices can aid in the development of more effective community based strategy for managing wasting among children with reduced risk of childhood obesity and long-term metabolic disease in India.

## Materials and methods

Children aged between 6 and 59 months with uncomplicated SAM using WHZ < -3 criteria included in this analysis are part of a larger multicentric, controlled study [20]. Children from the four intervention Indian states were recruited in the study soon after their enrolment in the community-based SAM management program while children from the control area were recruited from one Indian state where there is no community-based SAM management program. All the study participants were recruited during the year 2021 after obtaining written informed consent from their parents/guardians. Participation of children was voluntary and children could withdraw at any time without further obligations. Each child was assigned a unique identification number to ensure confidentiality.

Power calculations for the overall study indicated that a total sample of 83 children was needed per study state (expected cure rate of 50%, effect size of 25%, alpha error probability = 0.05, power = 0.80 and design effect = 1.5). Assuming an overall dropout-rate of 20%, the minimum sample size was determined to be 100 children per study state. In total, 659 children were recruited in the study during the baseline round. We achieved the minimum sample size of 100 in each study state.

During the baseline, anthropometric measurements (weight, height/length, mid-upper arm circumference) were taken alongside other socio-demographic information such as age, sex, mother’s age, mother’s years of education, caste, possession of different assets by the households and history of illness during the preceding 15 days on the day of survey. Body composition estimates for FM and FFM were also assessed via bioelectrical impedance analysis (BIA) using the InBody S10 machine, a lightweight portable scale that uses tetra-polar BIA with four electrodes at the arms and feet. Body composition estimates were taken from 504 children (111 children in lying position, 390 children in sitting position and 3 children in standing position) whose body weights were more than 5 kgs at the time of enrolment. Children wearing only light clothing were asked to lie down or sit or stand and electrodes were fitted to ensure optimal contact according to the device manufacturer’s instructions.

As per the manufacturer’s instructions, before conducting BIA assessment, it was ensured that the child remains in the sitting, standing or lying postures, in which the assessment was conducted, for about 10 minutes, so that body water may be dispersed evenly inside the body. The child’s arms did not touch the trunk part of body and were spread around 15-degree angle away from trunk. The child’s thighs did not touch each other, and legs were spread to shoulder width. When the assessment was conducted, we also made sure that the child’s bare feet do not touch the floor by putting a mat that does not conduct electricity. The machine was placed on a flat surface. Before the assessment, the child’s hands and feet were wiped with water to make them wet to get an accurate result. Any metallic devices which the child was wearing were removed and it was ensured the child has not eaten anything, preferably at least 1 hour before the test. Parameters of weight, height/length and age were entered in the InBody S10 machine. The reports on body composition parameters such as FM and FFM were autogenerated by the machine.

The Weighing scale (SECA 354: Capacity: 20 kg, Graduation: 5 g < 10 kg > 10 g) was used to assess body weight, to the nearest 0.1 kg of all children. Each child was measured twice. If the difference between first and second measurements were more than 100 g, then the third measurement was taken. The average of the two nearest measurements was taken as final weight of the child. Body length was taken for children aged 6 months to 2 years to the nearest 0.1 cm with each child lying straight on the infantometer (SECA 417: Measuring range: 10-100 cm/4–39 in., Graduation: 1 mm). Body height was taken for children aged above 2 years to the nearest 0.1 cm with each child standing with back erect and shoulders against a stadiometer (SECA 213: Measuring range: 20-205 cm/8–81 in., Graduation: 1 mm). Length or height of each child was also measured twice. If the difference in two measurements were found more than 0.7 cm then the third measurement was taken. The average of two nearest measurements were taken as final length/height of the child. Mid-upper arm circumference (MUAC) was taken of all children using non-stretchable fibre glass MUAC tape (Range upto 26.5 cm graduated with 1 mm, colour coded; Red: from 0 to 11.5 cm, Yellow: from 11.5 to 12.5 cm and Green: from 12.5 to 26.5 cm). Sex-specific weight-for-height, height-for-age, weight-for-age, body mass index (BMI) and MUAC z-scores were then computed from the World Health Organization (WHO) growth reference data [21]. The training and anthropometric standardisation exercise with the data collection staff and quality control measures were described elsewhere [20].

FM and FFM were taken as body composition parameters. As age was different of children included in the study, body composition parameters were adjusted for length/height to compute Fat Mass Index (FMI) and Fat Free Mass Index (FFMI). FMI and FFMI were taken as main body composition outcomes for the analyses [22].

Asset ownership (ownership of 25 durable assets such as electricity, mattress, pressure cooker, chair, table, cot/bed, electric fan, radio/transistor, television, sewing machine, mobile phone, landline phone, internet, computer, refrigerator, air-conditioner/cooler, washing machine, watch/clock, bicycle, motorcycle/scooter, animal-drawn cart, car, water pump, thresher and tractor) was measured with an index constructed using principal component analysis (PCA). Accordingly, a categorical variable for the household wealth index from 1 to 5 was created with 1 is categorised as Household Wealth Quintile 1 and was considered as poorest quintile and 5 was categorised as Household Wealth Quintile 5 as the least poor/richest quintile. Social/Caste status of the studied children were divided into two groups: Scheduled Tribe (ST) or Scheduled Caste (SC) and non-Scheduled Tribe/Scheduled Caste categories. The STs and SCs are officially designated groups of people and among the most disadvantaged socio-economic groups in India. Age of the studied children were divided into two groups: children aged 6–23 months and children aged 24–59 months.

The analyses include descriptive statistics (proportion and mean) to describe characteristics of the sample. Comparison of mean/average for anthropometry and body composition indices with age-group and sex-group were done using t-tests while with household wealth index using ANOVA test. Individual bivariate associations of anthropometric parameters (WHZ, WAZ, HAZ, BMIZ and MUACZ) with body composition parameters (FMI and FFMI) were assessed using Pearson Correlation analysis. Multiple linear regression models separately with WHZ, WAZ, HAZ,BMIZ and MUACZ as dependent variables were conducted to test their associations with each of these separate body composition estimates (FMI and FFMI) after adjusting for child’s age and sex, household wealth index and social/caste status. Associations of children with multiple deficits {both SAM (WHZ < -3 only criteria) and Severely Stunted (HAZ < -3)} separately with FMI and FFMI were tested to assess whether body composition indices are different in children with both severe wasting and severe stunting compared to children with severe wasting only. All statistical tests were performed using SPSS® 26 (IBM Corporation; Armonk, NY, USA) with statistical significance defined as *p* < 0.05.

## Results

### Descriptive characteristics of children

In total 659 children were enrolled in the study. Among all the children enrolled 46% children were from the age-group of 6–23 months ranging from 66.3% in Jharkhand to 35.1% in Chhattisgarh. Remaining children were from the age-group of 24–59 months. Of them 56.6% of enrolled children were male ranging from 62.6% in MP to 52.1% in Jharkhand. Around one-third (28.3%) of mothers did not receive any formal education and only around 10% of mothers receive more than 10 years of education. 65.1% of enrolled children were belonged to either SC or ST communities ranging from 86.5% in Jharkhand to 43.1% in Madhya Pradesh. About 51% of households were using toilet which was as high as 76.9% in Madhya Pradesh and as low as 12.5% in Odisha. Similarly, 87.7% households had provision of safe drinking water and 85.2% of households were using soap with water for handwashing after toilet. While caregivers of 54.5% of children reported illness of their enrolled child in the last 15 days ranging from 78.2% in Chhattisgarh to 28.2% in Jharkhand (Table 1).

**Table 1:** Descriptive characteristics

	All	Madhya Pradesh	Telangana	Odisha	Chhattisgarh	Jharkhand
**Age Group**	n (%)	n (%)	n (%)	n (%)	n (%)	n (%)
6-23 m	303 (46.0)	50 (38.2)	44 (39.6)	54 (45.0)	47 (35.1)	108 (66.3)
24-59 m	356 (54.0)	81 (61.8)	67 (60.4)	66 (55.0)	87 (64.9)	55 (33.7)
**Gender**						
Male	373 (56.6)	82 (62.6)	67 (60.4)	68 (56.7)	71 (53.0)	85 (52.1)
Female	286 (43.4)	49 (37.4)	44 (39.6)	52 (43.3)	63 (47.0)	78 (47.9)
**Mother’s Education**						
No Education	186 (28.3)	16 (12.3)	41 (36.9)	55 (45.8)	16 (12)	58 (35.6)
1–5 years	133 (20.2)	26 (20)	22 (19.8)	28 (23.3)	29 (21.8)	28 (17.2)
6–8 years	153 (23.3)	40 (30.8)	13 (11.7)	16 (13.3)	51 (38.3)	33 (20.2)
9–10 years	119 (18.1)	36 (27.7)	21 (18.9)	16 (13.3)	19 (14.3)	27 (16.6)
More than 10 years	66 (10)	12 (9.2)	14 (12.6)	5 (4.2)	18 (13.5)	17 (10.4)
**Caste_Tribe**						
SC/ST	428 (65.1)	56 (43.1)	31 (27.9)	89 (74.2)	111 (83.5)	141 (86.5)
Non SC/ST	229 (34.9)	74 (56.9)	80 (72.1)	31 (25.8)	22 (16.5)	22 (13.5)
**Use of Toilet**	335 (51)	100 (76.9)	68 (61.3)	15 (12.5)	97 (72.9)	55 (33.7)
**Safe Drinking Water Source**	576 (87.7)	122 (93.8)	111 (100)	120 (100)	122 (91.7)	101 (62)
**HW with Soap after Toilet**	560 (85.2)	118 (90.8)	79 (71.2)	103 (85.8)	115 (86.5)	145 (89)
**History of Illness last 15 days**	358 (54.5)	65 (50)	65 (58.6)	78 (65)	104 (78.2)	46 (28.2)

### Comparison of anthropometric and body composition parameters for children from different age groups, sex groups and household wealth index groups

Table 2 compares anthropometric (WHZ, WAZ, HAZ, BMIZ and MUACZ) and body composition indices (FMI and FFMI) of younger (children aged 6–23 months) and older (children aged 24–59 months) children. FFMI (*p*-value< 0.001) was significantly higher while WAZ (p-value = 0.02) and HAZ (p-value = 0.03) were significantly lower among younger children compared to older children. No significant differences were found in other anthropometric (WHZ, BMIZ and MUACZ) and body composition (FMI) indices between the two age groups of children.

**Table 2:** Comparison of anthropometric and body composition indices among younger and older children

Baseline Characteristics	Children Aged 6–23 Months			Children Aged 24–59 Months			t statistics
	N	Mean	SD	N	Mean	SD	
Fat Mass Index (FMI)	200	1.01	1.06	304	0.86	0.84	−1.69, 95% CI: −0.31 to 0.02, *p*-value = 0.09
Fat Free Mass Index (FFMI)	200	11.42	2.13	304	10.73	2.31	**− 3.39, 95% CI: −1.09 to − 0.29,** ***p*-value = 0.001**
WHZ	299	− 3.40	0.41	358	−3.40	0.39	0.03, 95% CI: − 0.06 to 0.06, p-value = 0.98
WAZ	299	−3.81	0.73	358	−3.67	0.63	**2.45, 95% CI: 0.03 to 0.24, p-value = 0.02**
HAZ	299	− 2.76	1.26	358	−2.55	1.12	**2.22, 95% CI: 0.02 to 0.39, p-value = 0.03**
BMIZ	299	−3.18	0.50	358	−3.15	0.46	0.93, 95% CI: −0.04 to 0.11, p-value = 0.35
MUACZ	299	−2.09	6.94	358	−2.61	2.77	−1.29, 95% CI: −1.30 to 0.27, p-value = 0.20

When the above indices were compared for male and female children, FMI (*p*-value = 0.02) was significantly higher while WHZ (*p*-value< 0.001) and HAZ (*p*-value = 0.003) were significantly lower among male children compared to female children. No significant differences were observed in other anthropometric (WAZ, BMIZ and MUACZ) and body composition (FFMI) indices between the two sex groups of children (Table 3).

**Table 3:** Comparison of anthropometric and body composition indices among male and female children

	Male			Female			
Anthropometric and Body Composition Parameters	N	Mean	SD	N	Mean	SD	t statistics
Fat Mass Index (FMI)	291	1.00	1.04	213	0.81	0.75	**2.27, 95% CI: 0.03 to 0.35, p-value = 0.02**
Fat Free Mass Index (FFMI)	291	11.04	2.41	213	10.94	2.04	0.50, 95% CI: −0.30 to 0.50, p-value = 0.62
WHZ	373	− 3.46	0.43	284	−3.32	0.32	**−4.42, 95% CI: −0.20 to − 0.08, p-value < 0.001**
WAZ	373	− 3.76	0.68	284	− 3.70	0.69	−1.15, 95% CI: −0.17 to 0.04, p-value = 0.25
HAZ	373	−2.77	1.20	284	−2.49	1.16	**−2.97, 95% CI: −0.46 to − 0.09, p-value = 0.003**
BMIZ	373	−3.17	0.52	284	−3.15	0.42	−0.47, 95% CI: − 0.09 to 0.06, p-value = 0.64
MUACZ	373	−2.11	6.75	284	−2.73	0.69	1.53, 95% CI: −0.17 to 1.41, p-value = 0.13

Anthropometric and body composition parameters were further compared for children from different household wealth index groups. FFMI (*p*-value = 0.02) was significantly higher among households from poorer wealth quintiles compared to richer wealth quintiles. However, HAZ (p-value< 0.001) and WAZ (p-value< 0.001) were significantly lower among households from poorer wealth quintiles compared to richer wealth quintiles. No significant differences were observed in other anthropometric (WHZ, BMIZ and MUACZ) and body composition (FMI) indices between the five wealth index groups (Table 4).

**Table 4:** Comparison of anthropometry and body composition indices of different household wealth quintile groups

Parameters	Household Wealth Quintile	N	Mean	SD	95% CI for Mean		ANOVA Statistics
					Lower Bound	Upper Bound	
**FMI**	Household Wealth Quintile 1	97	1.10	1.06	0.89	1.31	F = 2.21, *p*-value = 0.07
	Household Wealth Quintile 2	96	0.74	0.69	0.60	0.88	
	Household Wealth Quintile 3	102	0.88	0.82	0.72	1.05	
	Household Wealth Quintile 4	108	1.01	1.10	0.80	1.22	
	Household Wealth Quintile 5	101	0.85	0.89	0.68	1.03	
**FFMI**	Household Wealth Quintile 1	97	11.18	1.58	10.86	11.50	**F = 2.89,** ***p*-value = 0.02**
	Household Wealth Quintile 2	96	11.55	0.85	11.38	11.72	
	Household Wealth Quintile 3	102	10.91	2.36	10.45	11.37	
	Household Wealth Quintile 4	108	10.90	2.39	10.44	11.36	
	Household Wealth Quintile 5	101	10.51	3.21	9.88	11.14	
**HAZ**	Household Wealth Quintile 1	131	−2.96	1.26	−3.18	−2.74	**F = 7.28,** ***p*-value < 0.001**
	Household Wealth Quintile 2	132	−2.84	1.20	−3.04	−2.63	
	Household Wealth Quintile 3	131	−2.71	1.06	−2.89	−2.53	
	Household Wealth Quintile 4	132	−2.44	1.05	−2.63	−2.26	
	Household Wealth Quintile 5	131	−2.29	1.26	−2.51	−2.08	
**WAZ**	Household Wealth Quintile 1	131	−3.94	0.69	−4.06	−3.83	**F = 8.58,** ***p*-value < 0.001**
	Household Wealth Quintile 2	132	−3.83	0.69	−3.95	−3.72	
	Household Wealth Quintile 3	131	−3.76	0.64	−3.87	−3.65	
	Household Wealth Quintile 4	132	−3.61	0.61	−3.72	−3.51	
	Household Wealth Quintile 5	131	−3.52	0.71	−3.64	−3.39	
**WHZ**	Household Wealth Quintile 1	131	−3.45	0.37	−3.51	−3.38	F = 0.99, *p*-value = 0.42
	Household Wealth Quintile 2	132	−3.41	0.43	−3.48	−3.33	
	Household Wealth Quintile 3	131	−3.41	0.43	−3.48	−3.33	
	Household Wealth Quintile 4	132	−3.40	0.40	−3.46	−3.33	
	Household Wealth Quintile 5	131	−3.35	0.37	−3.42	−3.29	
**BMIZ**	Household Wealth Quintile 1	131	−3.20	0.49	−3.29	−3.12	F = 0.34, *p*-value = 0.85
	Household Wealth Quintile 2	132	−3.14	0.51	−3.23	−3.06	
	Household Wealth Quintile 3	131	−3.14	0.47	−3.22	−3.06	
	Household Wealth Quintile 4	132	−3.16	0.47	−3.24	−3.08	
	Household Wealth Quintile 5	131	−3.16	0.45	−3.24	−3.08	
**MUACZ**	Household Wealth Quintile 1	131	−2.93	0.64	−3.04	−2.82	F = 1.72, *p*-value = 0.15
	Household Wealth Quintile 2	132	−3.01	0.71	−3.13	−2.88	
	Household Wealth Quintile 3	131	−2.22	7.31	−3.49	−0.96	
	Household Wealth Quintile 4	132	−2.08	6.03	−3.12	−1.04	
	Household Wealth Quintile 5	131	−1.64	6.29	−2.73	− 0.55	

### Bivariate associations of anthropometric and body composition indices

Correlation between anthropometric parameters (WHZ, WAZ, HAZ, MUACZ and BMIZ) and body composition parameters [FMI and FFMI] shows that WHZ (*p*-value = 0.006) and BMIZ (p-value = 0.02) were positively correlated with FFMI while MUACZ was negatively corelated with FFMI (p-value< 0.001). This means that any increase in WHZ and BMIZ also increases FFMI. Increase in MUACZ decreases FFMI. There were no significant associations observed with FMI to any of the anthropometric indices (Table 5).

**Table 5:** Correlation of anthropometry and body composition indices

Baseline Parameters	WHZ		HAZ		WAZ		MUACZ		BMIZ	
	Corr. Coeff.	***p***-value	Corr. Coeff.	***p***-value	Corr. Coeff.	***p***-value	Corr. Coeff.	***p***-value	Corr. Coeff.	***p***-value
**Fat Mass Index (FMI)**	− 0.02	0.74	− 0.05	0.28	− 0.03	0.45	0.06	0.20	0.01	0.90
**Fat Free Mass Index (FFMI)**	**0.12**	**0.006**	0.04	0.33	0.85	0.06	**−0.20**	**< 0.001**	**0.11**	**0.02**

Multiple linear regression models separately with WHZ, WAZ, HAZ, MUACZ and BMIZ as dependent variables show that there were significant positive associations between WHZ, WAZ and BMIZ with FFMI. This implies that, increase in FFMI increase WHZ, WAZ and BMIZ. MUACZ was negatively associated with FMI and FFMI which implies that increase in FMI and FFMI decrease MUACZ. WHZ, WAZ and HAZ were significantly lower among male children compared to female children. MUACZ was significantly lower among older children (children aged 24–59 months) compared to younger children (children aged 6–23 months). Compared to richest households, WHZ, WAZ and HAZ were significantly lower among poorer households (Table 6).

**Table 6:** Multivariate association of anthropometry and body composition indices

Z-Scores	WHZ			WAZ			HAZ			BMIZ			MUACZ		
Parameters	β Coeff.	SE	95% CI	β Coeff.	SE	95% CI	β Coeff.	SE	95% CI	β Coeff.	SE	95% CI	β Coeff.	SE	95% CI
(Constant)	−3.48***	0.1	−3.67, −3.29	− 3.79***	0.17	−4.12, − 3.46	−2.49***	0.30	−3.07, −1.91	− 3.34***	0.12	−3.57, − 3.11	3.73***	1.30	1.18, 6.27
Age-group (24_59m = 1, 6_23m = 0)	0.001	0.04	− 0.07, 0.07	0.06	0.06	− 0.05, 0.18	0.12	0.11	−0.09, 0.33	0.01	0.04	−0.07, 0.09	−1.28***	0.46	−2.18, −0.37
Sex (Male = 1, Female = 0)	−0.15***	0.03	−0.22, − 0.09	− 0.13**	0.06	−0.24, − 0.02	− 0.40***	0.10	−0.60, − 0.20	−0.01	0.04	−0.09, 0.07	0.56	0.44	−0.31, 1.43
Caste_tribe (ST/SC = 1, Others = 0)	−0.05	0.04	−0.12, 0.03	−0.04	0.06	−0.16, 0.08	− 0.30	0.11	− 0.25, 0.19	−0.05	0.04	−0.14, 0.04	0.03	0.49	−0.92, 0.99
Household Wealth Quintile 1	−0.11*	0.06	−0.21, 0.01	−0.43***	0.09	−0.61, − 0.25	− 0.71***	0.17	−1.04, − 0.38	−0.004	0.07	−0.13. 0.13	− 0.41	0.74	− 1.86, 1.04
Household Wealth Quintile 2	−0.08	0.06	−0.19, 0.03	− 0.36***	0.09	− 0.54, − 0.17	− 0.62***	0.17	−0.95, − 0.29	0.02	0.07	− 0.11, 0.15	−0.36	0.73	−1.80, 1.08
Household Wealth Quintile 3	−0.04	0.05	−0.15, 0.06	−0.26***	0.09	−0.44, − 0.08	− 0.45***	0.16	−0.77, − 0.14	0.03	0.06	− 0.10, 0.15	0.52	0.71	−0.86, 1.91
Household Wealth Quintile 4	−0.06	0.05	−0.16, 0.04	−0.15*	0.09	−0.32, 0.03	− 0.24	0.16	− 0.55, 0.07	−0.01	0.06	−0.14, 0.11	0.57	0.69	−0.78, 1.93
FMI	0.01	0.02	−0.03, 0.04	−0.003	0.03	−0.06, 0.06	− 0.02	0.05	− 0.13, 0.09	0.01	0.02	−0.03, 0.05	− 0.48**	0.24	− 0.95, − 0.01
FFMI	0.02***	0.01	0.01, 0.04	0.04***	0.01	0.01, 0.06	0.04*	0.02	−0.002, 0.09	0.02**	0.01	0.00, 0.04	−0.48***	0.10	−0.68, − 0.29

***p-value < 0.01, **p-value < 0.05, *p-value < 0.10

SAM with severe stunting shows significantly lower FFM (p-value< 0.001) compared to those who were SAM only however, there is no significant association was observed between these two with respect to FM (Table 7).

**Table 7:** Comparison of anthropometric and body composition indices among children with both severe wasting and severe stunting versus children with severe wasting only

Baseline Characteristics	Children with Severe Wasting Only			Children with both Severe Wasting & Severe Stunting			F statistics
	N	Mean	SD	N	Mean	SD	
Fat Mass (FM)	325	0.64	0.63	162	0.60	0.57	F-value = 0.53, *p*-value = 0.47
Fat Free Mass (FFM)	325	8.09	1.80	162	6.87	1.52	**F-value = 54.20,** ***p*-value < 0.001**

## Discussion

The study was conducted among children with SAM using WHZ < -3 criteria enrolled in an ongoing multicentric longitudinal study. In the present paper, we assessed associations between anthropometric and body composition parameters (FMI and FFMI) among them. We have found that baseline estimates of anthropometric parameters were associated with body composition parameters. Children with higher FFMI were found to have higher WHZ, WAZ and BMIZ while children with lower FMI and FFMI had higher MUAZ. FFMI was significantly higher among younger age group of children (6-23 m) and among children from poorer households. FMI was significantly higher among male children. WAZ and HAZ were significantly lower among younger age group of children (6-23 m) while WHZ and HAZ were significantly lower among male children. HAZ and WAZ were significantly lower among children from poorer households.

The associations between impaired nutritional growth and reduced FMI and FFMI in children have been found in other studies conducted in different LMICs. In Ethiopia, it was found that non-oedematous malnourished children had reduced FFM compared to relatively healthy children [23]. Wasting and stunting have been shown to be interrelated conditions, both associated with increased mortality and affecting the same population and often the same child [24, 25]. The effect on mortality is especially strong when both are present [26]. In our data, SAM with severely stunted children had significantly lower FFM compared to those who were SAM only, indicating that lower FFM is associated with linear growth retardation. There was no difference in FM between SAM with severely stunting and SAM only children. These findings are consistent with those of other studies, which have reported deficits in FFM in stunted children, sometimes accompanied by a relative preservation of FM [27]. This was also found in the study conducted in Burkina Faso [22]. In the study from Cambodia, similar to our findings, FFM declined strongly in proportion with the degree of linear growth retardation. Crude associations between FM and the magnitude of growth faltering were found weaker; similar to our study findings [28]. In this study, therefore, tissue accretion was proportional to linear growth, with FFM most affected in absolute terms. Overall, studies of stunted children have relatively consistently showed deficits in FFM. Whether stunting is causally associated with later adiposity remains less clear and can be studied later.

A little is known about the relative importance of FM and FFM for survival in malnourished children. Fat provides energy for both body functions and the immune system, while muscle provides proteins which is needed for the inflammation response and immune system [29]. In the long run, low FFM is a risk factor of non-communicable diseases [30]. It is not possible, at this point, to infer causality in these associations given the cross-sectional design of the study however, after the end of the ongoing study, we might able to show changes in FM and FFM with changes in anthropometric parameters. Also, as the ongoing study is an experimental study, we might also be able to show differential changes in FM and FFM among children who have recovered or those who failed to recover. Final results of the ongoing study may additionally help us defining the recovery criteria based on body composition parameters alongside currently used anthropometric parameter (WHZ > = − 2).

Although methodological challenges remain, there is a growing interest for study on this topic because nutrition community is now looking beyond survival of children in the short run to helping them thrive in the long run. The strength of the study is that a large group of children are included in the study. Additionally, the BIA technique used in the study provides objective data on the body composition parameters such as FM and FFM. We also looked at several significant interactions including age, sex and socio-economic status. As this is a cross-sectional study design, we cannot exclude the possibility of selection bias. Also, because of the cross-sectional study design, causality cannot be inferred. However, this study is meant as a baseline analysis of an on-going multicentric study and not an endpoint in itself. The follow-up assessments at two additional time points will allow us to provide a more accurate picture of associations between anthropometric parameters and body composition and may help establishing causal relationship.

## Conclusion

The present study shows the association between malnutrition and body composition and found that all forms of undernutrition appear to adversely impact FFM and FM. FFM is found to be low in proportion to linear growth retardation. As the agenda in child undernutrition not only have the objective of making children survive but also help them to thrive, body composition study becomes increasingly important.

## Data Availability

All data and materials will be available for any further analyses. Please contact Dr. Rajesh Kumar Sinha Email: cmarajesh@gmail.com for data from this study.

## References

[1] Black RE, Victora CG, Walker SP, Bhutta ZA, Christian P, de Onis M (2013). Maternal and child undernutrition and overweight in low-income and middle-income countries. Lancet.

[2] Waterlow JC (1973). Note on the assessment and classification of protein-energy malnutrition in children. Lancet.

[3] Initiatives D (2018). 2018 global nutrition report: shining a light to spur action on nutrition.

[4] Wells JCK. Body composition of children with moderate and severe undernutrition and after treatment: a narrative review. BMC Med. 2019;17(1):215. Published 2019 Nov 25. 10.1186/s12916-019-1465-8 10.1186/s12916-019-1465-8 PMC6878632 31767002

[5] Holmes CJ, Racette SB. The Utility of Body Composition Assessment in Nutrition and Clinical Practice: An Overview of Current Methodology. Nutrients. 2021;13(8):2493. Published 2021 Jul 22. 10.3390/nu13082493 10.3390/nu13082493 PMC8399582 34444653

[6] Wells JC (2010). The evolutionary biology of human body fat: thrift and control.

[7] Lord G (2002). Role of leptin in immunology. Nutr Rev.

[8] Bartz S, Mody A, Hornik C, Bain J, Muehlbauer M, Kiyimba T (2014). Severe acute malnutrition in childhood: hormonal and metabolic status at presentation, response to treatment, and predictors of mortality. J Clin Endocrinol Metab.

[9] Njunge JM, Gwela A, Kibinge NK, Ngari M, Nyamako L, Nyatichi E (2019). Biomarkers of post-discharge mortality among children with complicated severe acute malnutrition. Sci Rep.

[10] Bazzano AN, Potts KS, Bazzano LA, Mason JB. The life course implications of ready to use therapeutic food for children in low-income countries. Int J Environ Res Public Health 2017;14(4):403. Published 2017 Apr 11. 10.3390/ijerph14040403. 10.3390/ijerph14040403 PMC5409604 28398257

[11] Fabiansen C, Yameogo CW, Iuel-Brockdorf AS, Cichon B, Rytter MJH, Kurpad A (2017). Effectiveness of food supplements in increasing fat-free tissue accretion in children with moderate acute malutrition: a randomised 2 x 2 x 3 factorial trial in Burkina Faso. PLoS Med.

[12] Lelijveld N, Seal A, Wells JC, Kirkby J, Opondo C, Chimwezi E (2016). Chronic disease outcomes after severe acute malnutrition in Malawian children (ChroSAM): a cohort study. Lancet Glob Health.

[13] Bénéfce E, Garnier D, Simondon KB, Malina RM (2001). Relationship between stunting in infancy and growth and fat distribution during adolescence in Senegalese girls. Eur J Clin Nutr.

[14] Savanur MS, Ghugre PSBMI (2016). Body fat and waist-to-height ratio of stunted v. non-stunted Indian children: a case-control study. Public Health Nutr.

[15] Walker SP, Chang SM, Powell CA (2007). The association between early childhood stunting and weight status in late adolescence. Int J Obes.

[16] Pomeroy E, Stock JT, Stanojevic S, Miranda JJ, Cole TJ, Wells JC (2014). Stunting, adiposity, and the individual-level “dual burden” among urban lowland and rural highland Peruvian children. Am J Hum Biol.

[17] Kagura J, Feeley AB, Micklesfield LK, Pettifor JM, Norris SA (2012). Association between infant nutrition and anthropometry, and pre-pubertal body composition in urban south African children. J Dev Orig Health Dis.

[18] Cameron N, Wright MM, Griffiths PL, Norris SA, Pettifor JM (2005). Stunting at 2 years in relation to body composition a 9 years in African urban children. Obes Res.

[19] Martins PA, Hoffman DJ, Fernandes MT, Nascimento CR, Roberts SB, Sesso R, Sawaya AL (2004). Stunted children gain less lean body mass and more fat mass than their non-stunted counterparts: a prospective study. Br J Nutr.

[20] Kumar P, Sinha RK, Daniel A (2021). Effectiveness of community-based treatment programs for treatment of uncomplicated severe acute malnourished children aged 6–59 months using locally produced nutrient dense foods: protocol for a multicentric longitudinal quasi-experimental study. BMC Nutr.

[21] Cole TJ (2012). The development of growth references and growth charts. Ann Hum Biol.

[22] Fabiansen C, Cichon B, Yaméogo CW, Iuel-Brockdorf AS, Phelan KPQ, Wells JC, Ritz C, Filteau S, Briend A, Christensen VB, Ashorn P, Michaelsen KF, Shepherd S, Friis H. Publisher correction: association between admission criteria and body composition among young children with moderate acute malnutrition, a cross-sectional study from Burkina Faso. Sci Rep 2020 10(1):16914. 10.1038/s41598-020-73323-6. Erratum for: Sci Rep. 2020 Aug 6;10(1):13266. PMID: 33020533; PMCID: PMC7536289. 10.1038/s41598-020-73323-6 PMC7536289 33020533

[23] Girma NT (2014). Bioimpedance in severely malnourished children: an emerging method for monitoring hydration of children with severe acute malnutrition. Copenhagen: Department of Nutrition, exercise and sports, faculty of science.

[24] Skau JK, Touch B, Chhoun C, Chea M, Unni US, Makurat J (2015). Effects of animal source food and micronutrient fortification in complementary food products on body composition, iron status, and linear growth: a randomized trial in Cambodia. Am J Clin Nutr.

[25] Briend A, Khara T, Dolan C (2015). Wasting and stunting—similarities and diferences: policy and programmatic implications. Food Nutr Bull.

[26] Schoenbuchner SM (2019). The relationship between wasting and stunting: A retrospective cohort analysis of longitudinal data in Gambian children from 1976 to 2016. Am J Clin Nutr..

[27] Martins PA, Hoffman DJ, Fernandes MT, Nascimento CR, Roberts SB, Sesso R, Sawaya AL. Stunted children gain less lean body mass and more fat mass than their non-stunted counterparts: a prospective study. Br J Nutr 2004 Nov;92(5):819–825. 10.1079/bjn20041274. PMID: 15533271. 10.1079/bjn20041274 15533271

[28] Skau JKH (2019). Stunting, wasting and breast-feeding as correlates of body composition in Cambodian children at 6 and 15 months of age. Br J Nutr.

[29] Fabiansen C, Cichon B, Yaméogo CW, Iuel-Brockdorf AS, Phelan KPQ, Wells JC, Ritz C, Filteau S, Briend A, Christensen VB, Ashorn P, Michaelsen KF, Shepherd S, Friis H. Publisher correction: association between admission criteria and body composition among young children with moderate acute malnutrition, a cross-sectional study from Burkina Faso. Sci Rep 2020 10(1):16914. 10.1038/s41598-020-73323-6. Erratum for: Sci Rep. 2020 Aug 6;10(1):13266. 10.1038/s41598-020-73323-6 PMC7536289 33020533

[30] Reeds PJ, Fjeld CR, Jahoor F (1994). Do the diferences between the amino acid compositions of acute-phase and muscle proteins have a bearing on nitrogen loss in traumatic states?. J Nutr.

